# Carbohydrate quality, not quantity, linked to reduced colorectal cancer incidence and mortality in US populations: evidence from a prospective study

**DOI:** 10.1186/s12916-024-03325-y

**Published:** 2024-03-05

**Authors:** Yi Xiao, Ling Xiang, Yahui Jiang, Yunhao Tang, Haitao Gu, Yaxu Wang, Linglong Peng

**Affiliations:** 1 Department of Gastrointestinal Surgery, The Second Affiliated Hospital of Chongqing Medical University, No.288 Tianwen Avenue, Chongqing, 400010 Nan’an District China; 2 Department of Clinical Nutrition, The Second Affiliated Hospital of Chongqing Medical University, No.288 Tianwen Avenue, Chongqing, 400010 Nan’an District China

**Keywords:** Colorectal cancer, Cancer prevention, Epidemiology, Dietary pattern, Carbohydrate quality index, Low-carbohydrate diet

## Abstract

**Background:**

Carbohydrates have been implicated in colorectal cancer (CRC) risk, but the specific impact of carbohydrate quality and quantity on CRC susceptibility in US populations remains unclear.

**Methods:**

We followed 101,694 participants from Prostate, Lung, Colorectal, and Ovarian Cancer Screening Trial. The carbohydrate quality index (CQI) and low-carbohydrate diet score (LCDs) were used to evaluate the daily carbohydrate quality and quantity separately, where higher scores indicated greater adherence. Cox proportional hazards regression was used to compute HRs and 95% CIs for incident CRC and related death. Subgroup analyses were conducted to identify potential effect modifiers.

**Results:**

During follow-up, we documented 1085 incident cases of CRC, of whom 311 died from CRC. Individuals in the highest compared with the lowest quartiles of CQI had a lower CRC incidence (Q4 vs Q1: HR 0.80, 95% CI 0.67–0.96, *P*_trend_ = 0.012) and mortality (Q4 vs Q1: HR 0.61, 95% CI 0.44–0.86, *P*_trend_ = 0.004). The inverse association between CQI and CRC risk was observed for distal colon and rectum but not for proximal colon cancer. Regarding mortality, this association was only significant for rectum cancer. Subgroup analyses indicated this inverse association of CQI with CRC risk was only observed in participants with lower LCDs. No significant associations were found between LCDs and CRC incidence or mortality.

**Conclusions:**

Our findings suggest focusing on higher quality, rather than restricting the quantity, of carbohydrate consumption may be an effective approach to reduce the risk of CRC in the US population, particularly for distal colon and rectal cancers.

**Supplementary Information:**

The online version contains supplementary material available at 10.1186/s12916-024-03325-y.

## Background

Colorectal cancer (CRC) remains a major public health challenge in the USA, with over 150,000 estimated new cases and 50,000 related deaths projected in 2023 alone [1], making it the second and third most common cancer in women and men separately [2]. The substantial associated health and economic burden underscores the urgent need for effective prevention strategies to curb CRC incidence and mortality.

Among modifiable lifestyle factors, diet has been recognized as a major contributor to CRC risk, accounting for more than 40% of CRC incidence and mortality [3]. Notably, as major dietary energy sources that directly impact blood glucose and insulin levels in all individuals, carbohydrates have been identified as potential factors linked to CRC risk [4–6]. However, existing studies primarily examining overall carbohydrate quantity have produced conflicting findings [7–9]. For instance, an Iranian study revealed the positive association between carbohydrate amount and CRC incidence [7], while other cohort analyses using low-carbohydrate diet scores (LCDs) to assess overall carbohydrate intake have yielded diverse conclusions. One study found that animal-rich LCDs increased colon cancer risk [8], but another one found that plant-rich LCDs may improve outcomes in CRC patients [9]. Given these discrepancies, the focus has shifted towards considering the quality, rather than just the quantity, of carbohydrates in relation to cancer risk. Recently, the carbohydrate quality index (CQI) has been developed as a comprehensive measure of carbohydrate quality, incorporating multiple factors like dietary fiber content and glycemic index [10]. A previous small case–control study in Iranians indicated that higher CQI and LCDs were associated with a reduced CRC risk [11]. However, it did not assess potential interrelationships between these scores or conduct location-specific CRC risk analyses. Moreover, crucial aspects, such as the correlation between CQI, LCDs, and CRC mortality, have not been adequately examined in previous studies. Overall, considering the diverse demographics and dietary habits in different populations, it is crucial to examine the potential correlations between CQI, LCDs, and the incidence and mortality of CRC in the US population.

In this study, we conducted a large-scale, prospective investigation with the aim of filling crucial knowledge gaps and unraveling the significance of carbohydrate quality and quantity, assessed using CQI and LCDs, respectively, in relation to CRC outcomes among Americans aged 55–74 years. Additionally, further analyses focusing on different anatomical subsites of CRC were also performed to determine whether these observed associations varied by the anatomical location of tumors. Our study may hold significant promise in guiding the development of effective preventive strategies to address the considerable health and economic burden posed by CRC in the USA.

## Methods

### Study design

This is a prospective study of participants in the Prostate, Lung, Colorectal, and Ovarian (PLCO) Cancer Screening Trial, which is a large randomized clinical trial funded by the National Cancer Institute (NCI) between 1993 and 2001 [12]. Approximately 150,000 men and women aged 55–74 years were enrolled from 10 screening centers across the United States. Participants were randomly assigned to receive either routine medical care (control arm) or additional screening tests for prostate, lung, colorectal, and ovarian cancers (intervention arm) [13]. The trial protocol was approved by institutional review boards at the NCI and participating centers. The PLCO Screening Trial initiative was granted approval by the Institutional Review Board of the NCI as well as every screening center involved in the study, with explicit, informed, and written consent obtained from all participants. The details on the design of the PLCO trial, including power calculations and recruitment methods, have been extensively documented in prior publications [14, 15].

### Data collection and covariates assessment

The demographic and lifestyle data of individuals were collected at baseline via self-administered questionnaires (Baseline Questionnaire, BQ) as part of the PLCO trial. The main data used in our study included age, sex, race, occupation, education level, smoking habits, pack-years of cigarettes, body mass index (BMI) at baseline, history of aspirin use, diabetes, colorectal diverticulitis or diverticulosis, colorectal polyp, colon comorbidities (that is, Gardner’s syndrome, ulcerative colitis, Crohn’s disease or familial polyposis), and family history of CRC. BMI was calculated as weight (kg) divided by height squared (m^2^). Dietary intake data were collected using a validated 137-item Food Frequency Questionnaire (FFQ) called the Dietary History Questionnaire (DHQ), administered 3 years after enrollment in the PLCO trial. The DHQ assessed the portion size, frequency, and types of foods and supplements consumed by participants over the past year. The validity of DHQ was demonstrated through comparison with a 24-h dietary recall study (that is, the Eating at America’s Table Study) [16]. In that study, DHQ performed better than other commonly used FFQs like the Block and Willett questionnaires in assessing absolute nutrient intake [16]. In the present study, physical activity was defined as weekly time spent in moderate to vigorous activity, collected via a supplemental questionnaire (SQX).

### Population for analysis

**Fig. 1:**
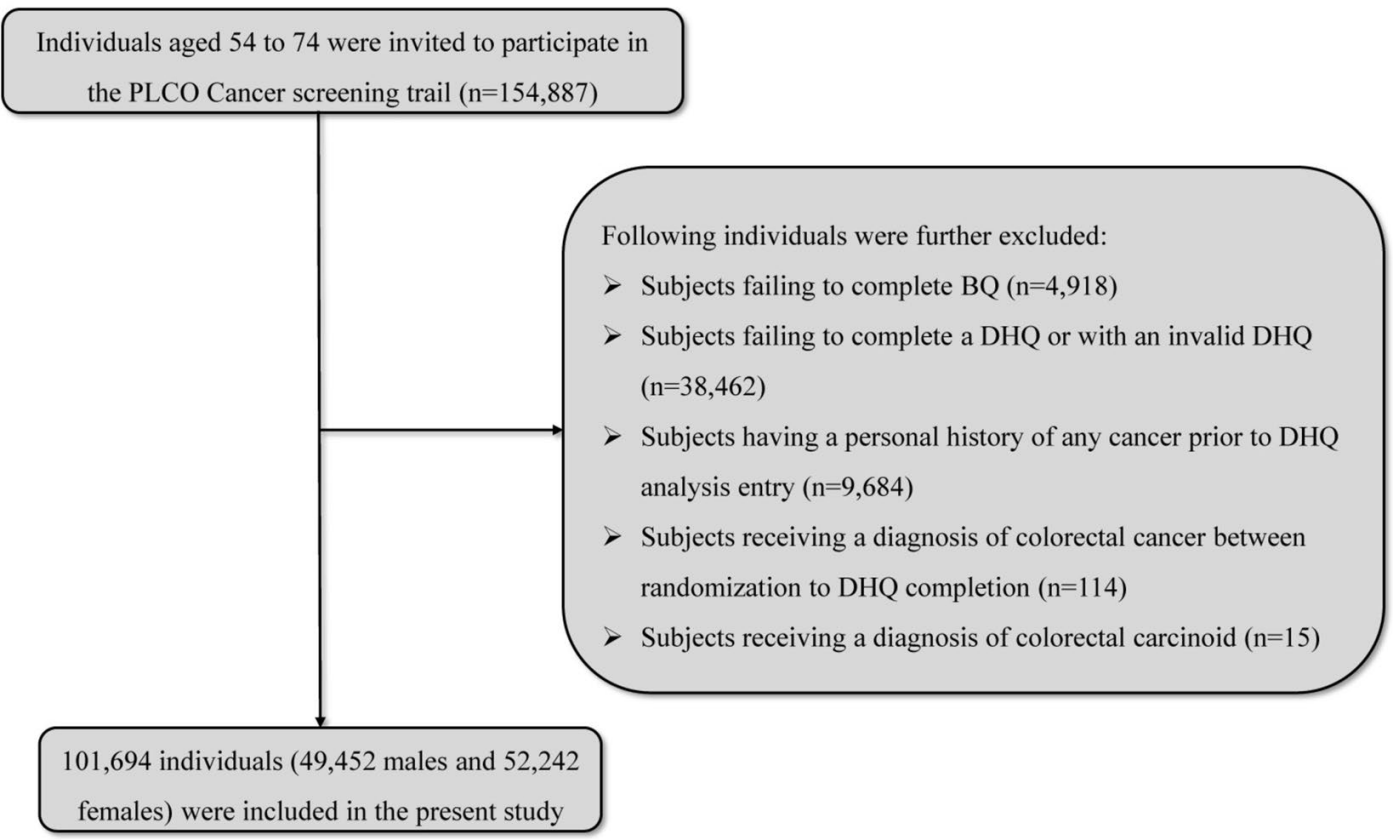
The flow chart of identifying eligible subjects. PLCO, Prostate, Lung, Colorectal, and Ovarian; BQ, Baseline Questionnaire; DHQ, Diet History Questionnaire

At baseline, we applied several exclusion criteria to determine the final analytic sample: (1) Did not return the BQ (*n* = 4918); (2) Had an invalid DHQ, defined as lacking completion date, confirmed death before completing, having ≥ 8 missing responses, or extreme calorie intake values (top, or bottom 1%) (*n* = 38,462); (3) Personal history of any cancer before DHQ completion (*n* = 9684); (4) Diagnosed with CRC between randomization and DHQ completion (*n* = 114); (5) Diagnosed with colorectal carcinoid (*n* = 15). Ultimately, our analytic sample consisted of 101,694 individuals (49,452 males and 52,242 females) (Fig. 1).

### Calculation of CQI and LCDs

In this study, the CQI and LCDs were calculated following the methods of Toledo et al. and Song et al., respectively, which was employed in the previous analyses [9, 10].

Specifically, CQI was calculated by summing the quintile scores of four equally-weighted criteria: dietary fiber intake (g/day, positively scored), glycemic index (GI, reversely scored), whole grain to total grain ratio (positively scored), and solid carbohydrate to total carbohydrate (solid + liquid carbohydrate) ratio (positively scored). For each criterion, participants were divided into quintiles and assigned scores from 1 to 5 based on the quintile value, except GI which was reversely scored. The total CQI score ranged from 4 to 20, with higher scores indicating better overall carbohydrate quality. Notably, the amount of liquid carbohydrates was calculated from the estimated consumption of sugar-sweetened beverages, and alcohol. Total grains are calculated by summing the intakes of whole grains, refined grains, and their products. In addition, the calculation methods related to GI have been described in detail in previous studies based on the PLCO trial [17, 18].

To calculate the LCDs, the intakes of carbohydrate, fat, and protein were first expressed as percentages of total energy consumption. These energy percentage values were then assigned into ranks from 0 to 10, with 11 equal-sized groups created ranging from the lowest percentage (rank 0) to the highest percentage (rank 10). However, the rank assignment direction differed by nutrient: for carbohydrates, lower percentages were assigned higher ranks (10 to 0), whereas for protein and fat, lower percentages were assigned lower ranks (0 to 10). By summing the nutrient ranks, with carbohydrate ranks reversely scored, the LCDs were generated on a scale of 0 to 30. Higher LCDs thus indicated lower carbohydrate but higher fat and protein intakes, representing more extreme low-carbohydrate dietary patterns.

Full details on the CQI and LCDs calculations and compositions are provided in Additional file 1: Table S1.

### Ascertainment of outcome events

In the PLCO trial, CRC cases were primarily ascertained through annual study update forms mailed to all surviving participants, which requested information on any new cancer diagnoses. Reported CRC cases were verified through medical record review using a standardized form, with study physicians confirming diagnoses in a blinded fashion. Vital status of participants was also tracked via the annual forms, with repeated attempts to contact non-responders. Additional mortality ascertainment involved routine checks of the National Death Index and death certificates using ICD-9 codes for causes of death.

CRC cases in the current study were categorized by anatomic subsites using International Classification of Disease (ICD)-O2 codes: proximal colon (C180-C185), distal colon (C186-C187), and rectum (C199-C209). When analyzing colorectum subsites, CRC coded as C188, C189, C212 and C218 were censored. It should be highlighted that the study’s main focus was on CRC incidence, while the secondary measure considered was CRC-related mortality.

### Statistical analysis

In this analysis, some covariate data were missing to varying degrees. For categorical variables with < 5% missing data, including education level, smoking status, history of aspirin uses, diabetes, colorectal diverticulitis or diverticulosis, colorectal polyp, colon comorbidities, and family history of CRC, missing values were imputed with mode value. For continuous variables with < 5% missing data, including BMI, and pack-years of cigarettes, median imputation was utilized. Multiple imputation methods were further applied to the physical activity level variable which had about 25% missing data [19]. Detailed information on the types of variables imputed and proportions missing were provided in Additional file 1: Table S2.

**Fig. 2:**
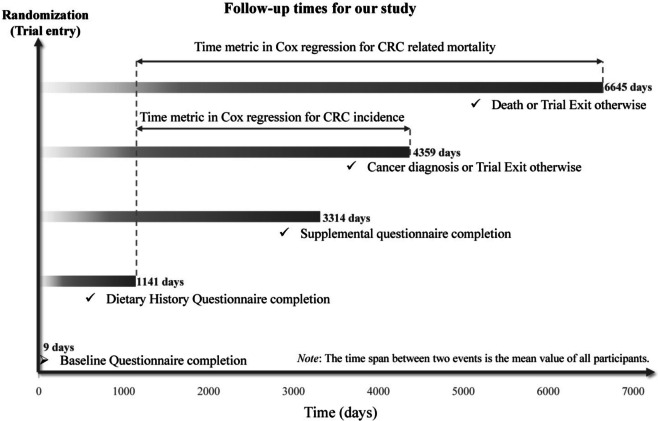
The timeline and follow-up scheme of our study. Notably, in our study, the baseline point was set at the date of diet history questionnaire completion

In the present study, time-to-CRC-event (diagnosis or related death) was defined as days from DHQ completion until CRC diagnosis or confirmation of CRC-related death. Hence, for primary outcome events, follow-up length was measured from the time of DHQ completion to the time of CRC diagnosis, death, lost, or December 31, 2009 (the end of cancer incidence follow-up), whichever happened first. For secondary outcome events, the end of mortality follow-up was 2018, which was detailed on the PLCO website (https://cdas.cancer.gov/learn/plco/early-qx/) (Fig. 2). Cox proportional hazards regression models were constructed to estimate the hazard ratios (HRs) and 95% confidence intervals (CIs) for the associations of the CQI and LCDs with outcome events, with the follow-up period as the time metric. The two mentioned scores were analyzed as continuous variables (HRs calculated per 1-standard deviation increment) and as categorical variables (in quartiles, with the first quartile being the referent group) in the Cox models. To assess potential linear trends, separate continuous variables were generated using the median CQI and LCDs within each quartile. The *P* value represents the significance of linear trends. Potential confounding variables were selected as established CRC risk factors or based on the clinical expertise of the investigators [20]. To mitigate the potential impact of confounding, these variables were incorporated in the Cox regression models of this study. Model 1 was adjusted for demographic characteristics, which included sex, age, race, and education level. Model 2 was further adjusted with lifestyle and clinical factors (BMI, physical activity level, smoking status, pack-years of cigarettes, alcohol consumption, history of colorectal diverticulitis or diverticulosis, colon comorbidities, colorectal polyp, aspirin use, diabetes, and family history of CRC), and total energy intake from diet. Considering the potential interaction between CQI and LCDs, Model 3 for each score adjusted for the covariates included in Model 2 plus the other score (CQI or LCDs). Restricted cubic spline models with knots at the 10th, 50th, and 90th percentiles were utilized to depict the trends of CRC incidence and mortality across the full range of the two scores [21]. The median value of the two scores was set as the reference, separately. Nonlinearity was tested by examining the null hypothesis that the regression coefficient for the second spline term equaled zero. In addition, the same analyses were conducted on anatomical subsites of CRC. The proportional hazards assumption was tested using Schoenfeld residuals and no violation was found [22].

Prespecified subgroup analyses were performed to explore potential effect modification of the associations between CQI and LCDs and CRC incidence by several key factors. Subgroups were defined by age, sex, smoking status, BMI, history of diabetes, aspirin use, dietary energy intake, and the score (CQI or LCDs). To avoid misleading subgroup effects, *P*-values for interaction were determined by comparing models with and without interaction terms prior to subgroup analyses. Moreover, *P*-values for trend across quartiles of the two scores were calculated within each subgroup using previously described methods. The purpose of these analyses was to assess the consistency and generalizability of the associations between the two scores and CRC outcomes in major population segments. Given the small number of CRC-related deaths in this study, we did not stratify analyses of CRC mortality in subgroup analyses.

Several sensitivity analyses were performed to enhance the robustness of the findings: (1) individuals with extreme energy intake (> 4000 kcal/day or < 500 kcal/day) or BMI (top or bottom 1%) were excluded; (2) outcome events within the initial 1 or 2 years of follow-up were excluded to assess potential reverse causation effects; (3) individuals with a history of colon comorbidities, colorectal polyps, or a family history of CRC were excluded, considering they are in high risk of CRC [20, 23]; (4) further adjusting for the carbohydrate intake (% E) directly instead of LCDs in the Model 3 of CQI analyses to determine whether this observed association was influenced by the amount of carbohydrate intake; (5) further adjusting for several dietary factors, including the energy-adjusted consumption of dietary calcium, calcium from supplements, energy-adjusted average daily red meat consumption, and total folate (combining dietary folate and folate from supplements). All statistical analyses were carried out using R software version 4.2.2, with two-tailed *P* < 0.05 as the level of statistical significance.

## Results

### Participant baseline features

In this study, the mean (standard deviation) was 12.0 (3.1) points for CQI, and 15.0 (7.1) points for LCDs. The two scores were negatively correlated (Pearson’s *R* =  − 0.062, *P* < 0.001). The baseline characteristics according to quartiles of CQI and LCDs are presented in Table 1. Participants in the highest CQI quartile had healthier lifestyles including more physical activity, lower cigarette pack-years, BMI, and alcohol consumption compared to those in the lowest quartile, whereas the highest LCDs quartile displayed an inverse pattern of less healthy lifestyles relative to the lowest quartile.

**Table 1:** Baseline characteristics of study population according to overall CQI and LCDs

		**CQI**		**LCD**s	
Characteristics	Overall	Quartile1 (Lowest)	Quartile 4 (Highest)	Quartile1	Quartile4
**Number of participants**	101,694	32,420	23,034	28,869	24,323
CQI	12.0 (3.1)	8.4 (1.6)	16.1 (1.2)	12.2 (3.2)	11.6 (3.1)
LCDs	15.0 (7.1)	15.5 (7.5)	14.2 (6.7)	6.4 (3.0)	24.3 (2.6)
**Demographic factors**					
Age	65.5 (5.7)	64.6 (5.6)	66.5 (5.7)	66.4 (5.9)	64.3 (5.4)
Female, %	51.4	52.6	50.8	56.8	44.2
***Racial/ethnic group, %***					
Non-Hispanic White	91.0	87.2	94.4	86.4	92.9
Non-Hispanic Black	3.3	4.5	2.1	5.0	2.5
Hispanic	1.5	1.4	1.6	1.4	1.8
Other race/ethnicity^a^	4.3	7.0	1.9	7.2	2.7
***Education level, %***					
Some college or less	63.8	67.2	60.2	63.4	65.6
College graduate	17.5	16.5	18.6	16.8	17.3
Postgraduate	18.6	16.3	21.2	19.8	17.0
**Lifestyle and clinical factors**					
Body mass index at baseline (kg/m^2^)	27.2 (4.8)	27.7 (5.0)	26.6 (4.6)	26.3 (4.5)	28.4 (5.0)
Physical activity level (min/week)	125.3 (108.3)	110.3 (101.1)	143.2 (114.7)	133.6 (113.8)	118.1 (103.7)
***Smoking status, %***					
Never	47.7	44.3	51.3	53.8	40.7
Current	9.2	12.6	6.2	6.6	12.9
Former	43.0	43.0	42.5	39.5	46.4
Pack-years of cigarettes	17.6 (26.6)	20.2 (28.4)	15.1 (24.6)	14.0 (23.8)	22.3 (29.6)
Alcohol consumption (g/day)	9.5 (25.3)	9.0 (24.2)	11.0 (31.8)	10.9 (38.0)	7.2 (12.0)
Family history of colorectal cancer, %	12.6	12.5	12.8	12.8	12.7
History of diverticulitis or diverticulosis, %	6.7	6.6	6.9	7.1	6.2
History of colon comorbidity, %	1.3	1.6	1.2	1.5	1.2
History of colorectal polyp, %	6.6	6.0	7.2	6.6	6.5
History of diabetes, %	6.7	7.1	6.3	4.7	9.8
Aspirin user, %	47.0	45.9	47.4	46.3	48.2
**Dietary factors**					
Total energy intake (kcal/d)	1738.5 (736.4)	1519.5 (654.2)	1979.2 (749.7)	1580.2 (701.6)	1920.2 (801.8)
Carbohydrate intake (% E)	52.0 (9.5)	50.3 (9.8)	54.2 (9.2)	61.6 (7.7)	42.1 (5.6)
Fat intake (% E)	31.7 (7.6)	33.1 (7.6)	29.9 (7.7)	24.4 (5.3)	39.5 (5.2)
Protein intake (% E)	15.4 (3.0)	14.8 (3.1)	16.0 (2.9)	13.5 (2.4)	17.6 (2.6)
Total fiber intake (g/day)	18.0 (8.5)	12.7 (5.3)	24.8 (8.9)	19.1 (9.3)	17.0 (8.1)
Glycemic index	53.6 (3.3)	55.3 (3.0)	51.4 (2.8)	53.5 (3.5)	53.5 (3.4)
Solid carbohydrates intake (g/day)	221.5 (91.5)	187.7 (83.0)	260.9 (87.8)	239.6 (99.1)	201.4 (86.0)
Liquid carbohydrates intake (g/day)	409.8 (476.4)	574.2 (582.6)	235.9 (264.3)	503.4 (558.8)	373.8 (489.2)
Whole grains intake (g/day)	61.1 (59.9)	32.0 (36.5)	96.0 (72.8)	74.0 (70.1)	46.4 (47.6)
Refined grains intake (g/day)	103.6 (93.6)	91.5 (86.4)	112.5 (96.4)	115.0 (111.7)	94.6 (80.0)
Dietary calcium intake (g/day)	749.5 (405.7)	548.6 (279.3)	999.4 (465.6)	679.8 (355.4)	796.3 (445.2)
Calcium intake from supplements (g/day)	256.7 (354.0)	228.7 (341.2)	289.7 (367.2)	289.4 (367.9)	217.1 (334.0)
Red meat intake (g/day)	61.5 (52.4)	61.8 (50.7)	57.7 (51.1)	33.4 (26.6)	100.5 (70.7)
Vegetables intake (g/day)	284.0 (184.4)	209.5 (126.2)	376.1 (223.3)	282.5 (207.7)	287.3 (174.7)
Fruits intake (g/day)	178.1 (150.6)	108.2 (91.8)	271.7 (187.6)	241.0 (192.2)	122.3 (102.4)
Total folate intake (g/day)^b^	599.3 (264.3)	497.3 (236.3)	710.8 (263.0)	624.0 (272.1)	575.4 (260.0)

*Abbreviations*: *CQI* carbohydrate quality index, *LCDs* low-carbohydrate diet score, *%E* the percentage of total energy intake. Values are shown as mean (standard deviation) unless stated otherwise

^a^ “Other race/ethnicity” refers to Asian, Pacific Islander, or American Indian

^b^Total folate including dietary folate and folate from supplements

During a mean of 8.81 years of follow-up, 1085 incident CRC cases were documented, among which 311 died from CRC over a longer follow-up period (15.07 years). These cases included 640 proximal colon cancers (181 deaths), 224 distal colon cancers (71 deaths), 199 rectal cancers (54 deaths), and 22 of unknown anatomical location (5 deaths).

### Association between CQI and CRC outcome events

**Fig. 3:**
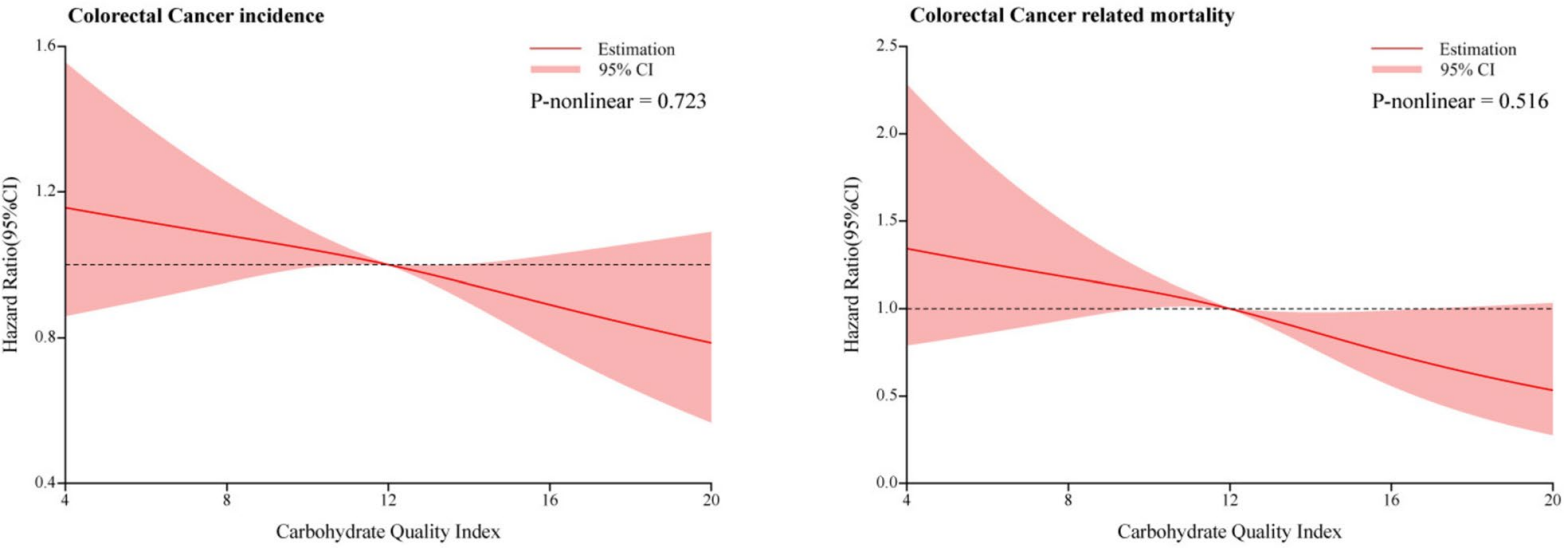
Nonlinear Dose–response analysis on the association of CQI with the risk of both colorectal cancer incidence and mortality. Hazard ratios were adjusted for age, sex, race, education levels, family history of colorectal cancer, history of colon comorbidities, history of diverticulitis or diverticulosis, history of colorectal polyp, history of diabetes, history of aspirin use, total energy intake, body mass index at baseline, smoking status, pack-years of cigarettes, alcohol consumption, physical activity level, and LCDs

The multi-model Cox regression analysis results of CQI and the incidence and mortality of CRC, including its subsites, were presented in Table 2 and 3, respectively. In comparison with participants in the lowest CQI quartile, those in the highest quartile had a significantly reduced incidence of CRC after adjusting for potential confounders (Model 3: HR _Quartile 4 *vs.* Quartile 1_: 0.80; 95% CI: 0.67, 0.96; *P* = 0.012 for trend). A similar result was observed in the mortality of CRC (Model 3: HR _Quartile 4 *vs.* Quartile 1_: 0.61; 95% CI: 0.44, 0.86; *P* = 0.004 for trend). Analyses modeling CQI as a continuous variable revealed significant inverse associations of higher CQI scores with CRC incidence (HR per SD increment: 0.93; 95% CI: 0.87, 0.99) and mortality (HR per SD increment: 0.84; 95% CI: 0.75, 0.95) in the Model 3. Restricted cubic spline regression models demonstrated linear dose–response relationships, whereby higher CQI scores were associated with lower risks of CRC incidence and mortality (all *P*-values for nonlinearity > 0.05; Fig. 3).

**Table 2:** Association between CQI and the CRC incidence according to main anatomic location

**Outcome**	**CQI**, HR (95% CI)					Continuous (per SD increment)
	Quartile 1 (lowest)	Quartile 2	Quartile 3	Quartile 4 (highest)	P for trend^a^	
Mean (SD) value of **CQI**	8.4 (1.6)	11.5 (0.5)	13.5 (0.5)	16.1 (1.2)		
Person-years	284,550	208,129	200,002	203,361		
**Overall**^b^						
Cases, *n*	373	249	238	225		
Incidence rate (95% CI)^c^	1.31 (1.18, 1.45)	1.20 (1.06, 1.35)	1.19 (1.05, 1.35)	1.11 (0.97, 1.26)		
Model 1^d^	1.00 (reference)	0.87 (0.74, 1.02)	0.85 (0.72, 1.00)	0.76 (0.65, 0.90)	0.001	0.91 (0.86, 0.97)
Model 2^e^	1.00 (reference)	0.89 (0.76, 1.05)	0.88 (0.75, 1.04)	0.81 (0.68, 0.96)	0.016	0.93 (0.87, 0.99)
Model 3^f^	1.00 (reference)	0.89 (0.76, 1.05)	0.88 (0.74, 1.04)	0.80 (0.67, 0.96)	0.012	0.93 (0.87, 0.99)
**Proximal colon**						
Cases, *n*	208	143	141	148		
Incidence rate (95% CI)^c^	0.73 (0.64, 0.84)	0.69 (0.58, 0.81)	0.70 (0.60, 0.83)	0.73 (0.62, 0.85)		
Model 1^d^	1.00 (reference)	0.88 (0.71, 1.09)	0.88 (0.71, 1.09)	0.87 (0.70, 1.08)	0.181	0.95 (0.87, 1.02)
Model 2^e^	1.00 (reference)	0.91 (0.74, 1.13)	0.94 (0.75, 1.17)	0.96 (0.76, 1.20)	0.664	0.98 (0.90, 1.07)
Model 3^f^	1.00 (reference)	0.91 (0.73, 1.13)	0.93 (0.74, 1.16)	0.94 (0.75, 1.18)	0.559	0.98 (0.90, 1.06)
**Distal colon**						
Cases, *n*	88	54	43	39		
Incidence rate (95% CI)^c^	0.31 (0.25, 0.38)	0.26 (0.20, 0.34)	0.21 (0.16, 0.29)	0.19 (0.14, 0.26)		
Model 1^d^	1.00 (reference)	0.83 (0.59, 1.16)	0.68 (0.47, 0.98)	0.60 (0.41, 0.88)	0.004	0.81 (0.71, 0.93)
Model 2^e^	1.00 (reference)	0.86 (0.61, 1.22)	0.72 (0.49, 1.05)	0.65 (0.43, 0.97)	0.023	0.84 (0.73, 0.97)
Model 3^f^	1.00 (reference)	0.86 (0.61, 1.22)	0.72 (0.49, 1.05)	0.65 (0.43, 0.97)	0.024	0.84 (0.73, 0.97)
**Rectum**						
Cases, *n*	71	47	49	32		
Incidence rate (95% CI)^c^	0.25 (0.2, 0.31)	0.23 (0.17, 0.30)	0.24 (0.19, 0.32)	0.16 (0.11, 0.22)		
Model 1^d^	1.00 (reference)	0.88 (0.61, 1.28)	0.95 (0.66, 1.37)	0.60 (0.40, 0.92)	0.030	0.89 (0.77, 1.02)
Model 2^e^	1.00 (reference)	0.88 (0.61, 1.28)	0.94 (0.64, 1.36)	0.59 (0.38, 0.91)	0.027	0.88 (0.76, 1.02)
Model 3^f^	1.00 (reference)	0.88 (0.61, 1.28)	0.93 (0.64, 1.36)	0.58 (0.38, 0.91)	0.027	0.88 (0.76, 1.02)

*Abbreviations*: *CQI* carbohydrate quality index, *CRC* colorectal cancer, *HR* hazard ratio, *CI* confidence interval, *SD* standard deviation

^a^Trend test was performed using the median value of each diet score quintile as a continuous variable

^b^Including 22 incident cases of CRC with an unknown anatomic location

^c^Incidence rate was calculated per 1000 person-years

^d^Model 1 was controlled with age (continuous), sex (male, female), race (non-Hispanic White, Non-Hispanic Black, Hispanic, other race/ethnicity), education levels (some college or less, college graduate, postgraduate)

^e^Model 2 was additionally controlled with a family history of colorectal cancer (no, yes or possibly), history of colon comorbidity (no, yes), history of diverticulitis or diverticulosis (no, yes), history of colorectal polyp (no, yes), history of diabetes (no, yes), history of aspirin use (no, yes), total energy intake (continuous), body mass index at baseline (continuous), smoking status (never, current, former), pack-years of cigarettes (continuous), alcohol consumption (continuous), and physical activity level (continuous)

^f^Model 3 was additionally controlled with a low-carbohydrate diet score (continuous)

**Table 3:** Association between CQI and the CRC mortality according to main anatomic location

**Outcome**	**CQI**, HR (95% CI)					Continuous (per SD increment)
	Quartile 1 (lowest)	Quartile 2	Quartile 3	Quartile 4 (highest)	P for trend^a^	
Mean (SD) value of **CQI**	8.4 (1.6)	11.5 (0.5)	13.5 (0.5)	16.1 (1.2)		
Person-years	488,250	354,591	342,265	347,391		
**Overall**^b^						
Cases, *n*	123	66	68	54		
Incidence rate (95% CI)^c^	0.25 (0.21, 0.30)	0.19 (0.15, 0.24)	0.20 (0.16, 0.25)	0.16 (0.12, 0.20)		
Model 1^d^	1.00 (reference)	0.68 (0.50, 0.92)	0.70 (0.52, 0.94)	0.52 (0.38, 0.72)	< 0.001	0.79 (0.70, 0.88)
Model 2^e^	1.00 (reference)	0.74 (0.55, 1.00)	0.79 (0.58, 1.07)	0.62 (0.44, 0.87)	0.004	0.84 (0.75, 0.95)
Model 3^f^	1.00 (reference)	0.74 (0.54, 1.00)	0.79 (0.58, 1.07)	0.61 (0.44, 0.86)	0.004	0.84 (0.75, 0.95)
**Proximal colon**						
Cases, *n*	61	45	41	34		
Incidence rate (95% CI)^c^	0.12 (0.10, 0.16)	0.13 (0.09, 0.17)	0.12 (0.09, 0.16)	0.10 (0.07, 0.14)		
Model 1^d^	1.00 (reference)	0.93 (0.63, 1.38)	0.85 (0.57, 1.26)	0.66 (0.43, 1.01)	0.054	0.85 (0.73, 0.99)
Model 2^e^	1.00 (reference)	1.02 (0.69, 1.50)	0.97 (0.64, 1.46)	0.79 (0.51, 1.24)	0.344	0.92 (0.78, 1.08)
Model 3^f^	1.00 (reference)	1.01 (0.68, 1.49)	0.96 (0.64, 1.44)	0.78 (0.50, 1.22)	0.300	0.91 (0.78, 1.07)
**Distal colon**						
Cases, *n*	33	12	11	15		
Incidence rate (95% CI)^c^	0.07 (0.05, 0.09)	0.03 (0.02, 0.06)	0.04 (0.02, 0.06)	0.04 (0.03, 0.07)		
Model 1^d^	1.00 (reference)	0.42 (0.21, 0.83)	0.45 (0.23, 0.87)	0.53 (0.28, 0.98)	0.019	0.76 (0.60, 0.96)
Model 2^e^	1.00 (reference)	0.45 (0.23, 0.91)	0.51 (0.26, 1.01)	0.64 (0.33, 1.22)	0.092	0.82 (0.64, 1.06)
Model 3^f^	1.00 (reference)	0.45 (0.23, 0.91)	0.52 (0.26, 1.02)	0.64 (0.33, 1.24)	0.099	0.82 (0.64, 1.06)
**Rectum**						
Cases, *n*	28	9	12	5		
Incidence rate (95% CI)^c^	0.06 (0.04, 0.08)	0.03 (0.01, 0.05)	0.04 (0.02, 0.06)	0.01 (0.01, 0.03)		
Model 1^d^	1.00 (reference)	0.43 (0.20, 0.91)	0.58 (0.29, 1.15)	0.23 (0.09, 0.61)	0.001	0.64 (0.49, 0.85)
Model 2^e^	1.00 (reference)	0.47 (0.22, 1.01)	0.66 (0.32, 1.33)	0.27 (0.10, 0.73)	0.006	0.69 (0.52, 0.93)
Model 3^f^	1.00 (reference)	0.47 (0.22, 1.01)	0.66 (0.32, 1.34)	0.27 (0.10, 0.73)	0.006	0.69 (0.52, 0.93)

*Abbreviations*: *CQI*, carbohydrate quality index; *CRC*, colorectal cancer; *HR*, hazard ratio; *CI*, confidence interval, *SD*, standard deviation

^a^Trend test was performed using the median value of each diet score quintile as a continuous variable

^b^Including 5 death cases related to CRC with an unknown anatomic location

^c^Incidence rate was calculated per 1000 person-years

^d^Model 1 was controlled with age (continuous), sex (male, female), race (non-Hispanic White, Non-Hispanic Black, Hispanic, other race/ethnicity), education levels (some college or less, college graduate, postgraduate)

^e^Model 2 was additionally controlled with a family history of colorectal cancer (no, yes or possibly), history of colon comorbidity (no, yes), history of diverticulitis or diverticulosis (no, yes), history of colorectal polyp (no, yes), history of diabetes (no, yes), history of aspirin use (no, yes), total energy intake (continuous), body mass index at baseline (continuous), smoking status (never, current, former), pack-years of cigarettes (continuous), alcohol consumption (continuous), and physical activity level (continuous)

^f^Model 3 was additionally controlled with a low-carbohydrate diet score (continuous)

In subsite analyses using multivariable Model 3, higher CQI scores were associated with decreased incidence of distal colon cancer (HR _Quartile 4 *vs.* Quartile 1_: 0.65; 95% CI: 0.43, 0.97; *P* = 0.024 for trend) and rectum cancer (HR _Quartile 4 *vs.* Quartile 1_: 0.58; 95% CI: 0.38, 0.91; *P* = 0.027 for trend), but not proximal colon cancer. Furthermore, this inverse association was also observed between CQI and rectal cancer mortality (HR _Quartile 4 *vs.* Quartile 1_: 0.27; 95% CI: 0.10, 0.73; *P* = 0.006 for trend) but not for other subsites.

In subgroup analyses stratified by major demographic and lifestyle factors, the inverse associations of CQI with CRC incidence were consistent and not modified by age, sex, smoking, BMI, aspirin use, diabetes history, energy intake, or LCDs (all *P*-interaction > 0.05; Additional file 1: Table S3). Sensitivity analyses that excluded some specific individuals or adjusted for additional covariates showed robust correlations between higher CQI and reduced incidence and mortality of CRC Additional file 1: Table S4–5).

### Association between LCDs and CRC outcome events

In multivariable Cox regression analyses, no significant associations were observed between LCDs and risks of overall CRC incidence (Model 3: HR _Quartile 4 *vs.* Quartile 1_: 0.92; 95% CI: 0.77, 1.10; *P* = 0.261 for trend; HR per SD increment: 0.96; 95% CI: 0.90, 1.02) or mortality (Model 3: HR _Quartile 4 *vs.* Quartile 1_: 1.02; 95% CI: 0.74, 1.42; *P* = 0.982 for trend; HR per SD increment: 0.98; 95% CI: 0.87, 1.10) when comparing extreme quartiles or modeling LCDs continuously (Table 4 and Additional file 1: Table S6). Similarly, in subsite analyses, LCDs were not significantly associated with the incidence or mortality of proximal colon, distal colon, or rectum cancers (all *P* > 0.05 for trend).

**Table 4:** Association between LCDs and the CRC incidence according to main anatomic location

**Outcome**	**LCD**s, HR (95% CI)					Continuous (per SD increment)
	Quartile 1 (lowest)	Quartile 2	Quartile 3	Quartile 4 (highest)	P for trend^a^	
Mean (SD) value of **LCDs**	6.4 (3.0)	13.0 (1.4)	18.0 (1.4)	24.3 (2.6)		
Person-years	257,597	219,185	208,982	210,276		
**Overall**^b^						
Cases, *n*	323	274	242	246		
Incidence rate (95% CI)^c^	1.25 (1.12, 1.40)	1.25 (1.11, 1.41)	1.16 (1.02, 1.31)	1.17 (1.03, 1.33)		
Model 1^d^	1.00 (reference)	1.02 (0.87, 1.20)	0.96 (0.81, 1.13)	1.01 (0.85, 1.20)	0.912	1.00 (0.94, 1.06)
Model 2^e^	1.00 (reference)	1.00 (0.85, 1.17)	0.92 (0.78, 1.09)	0.94 (0.79, 1.12)	0.360	0.96 (0.90, 1.03)
Model 3^f^	1.00 (reference)	0.99 (0.85, 1.17)	0.91 (0.77, 1.08)	0.92 (0.77, 1.10)	0.261	0.96 (0.90, 1.02)
**Proximal colon**						
Cases, *n*	206	163	133	138		
Incidence rate (95% CI)^c^	0.80 (0.70, 0.92)	0.74 (0.64, 0.87)	0.64 (0.54, 0.75)	0.66 (0.56, 0.78)		
Model 1^d^	1.00 (reference)	0.96 (0.78, 1.18)	0.85 (0.68, 1.05)	0.94 (0.75, 1.17)	0.338	0.97 (0.89, 1.05)
Model 2^e^	1.00 (reference)	0.93 (0.76, 1.15)	0.81 (0.65, 1.02)	0.87 (0.69, 1.09)	0.114	0.94 (0.86, 1.02)
Model 3^f^	1.00 (reference)	0.93 (0.76, 1.15)	0.81 (0.65, 1.01)	0.86 (0.68, 1.08)	0.104	0.93 (0.86, 1.02)
**Distal colon**						
Cases, *n*	55	57	61	51		
Incidence rate (95% CI)^c^	0.21 (0.16, 0.28)	0.26 (0.20, 0.34)	0.29 (0.23, 0.37)	0.24 (0.18, 0.32)		
Model 1^d^	1.00 (reference)	1.27 (0.87, 1.84)	1.43 (0.99, 2.06)	1.21 (0.82, 1.79)	0.238	1.06 (0.92, 1.21)
Model 2^e^	1.00 (reference)	1.24 (0.85, 1.80)	1.38 (0.95, 2.00)	1.13 (0.75, 1.69)	0.435	1.02 (0.89, 1.18)
Model 3^f^	1.00 (reference)	1.24 (0.85, 1.80)	1.35 (0.93, 1.96)	1.09 (0.72, 1.63)	0.570	1.01 (0.88, 1.16)
**Rectum**						
Cases, *n*	56	47	45	51		
Incidence rate (95% CI)^c^	0.22 (0.17, 0.28)	0.21 (0.16, 0.29)	0.22 (0.16, 0.29)	0.24 (0.18, 0.32)		
Model 1^d^	1.00 (reference)	0.97 (0.65, 1.43)	0.95 (0.64, 1.41)	1.05 (0.71, 1.54)	0.860	1.03 (0.89, 1.19)
Model 2^e^	1.00 (reference)	0.96 (0.65, 1.42)	0.93 (0.62, 1.39)	1.00 (0.67, 1.50)	0.961	1.01 (0.87, 1.17)
Model 3^f^	1.00 (reference)	0.96 (0.65, 1.42)	0.91 (0.61, 1.37)	0.98 (0.65, 1.47)	0.855	1.00 (0.86, 1.16)

*Abbreviations*: *CQI*, carbohydrate quality index; *CRC*, colorectal cancer; *HR*, hazard ratio; *CI*, confidence interval, *SD*, standard deviation

^a^Trend test was performed using the median value of each diet score quintile as a continuous variable

^b^Including 22 incident cases of CRC with an unknown anatomic location

^c^Incidence rate was calculated per 1000 person-years

^d^Model 1 was controlled with age (continuous), sex (male, female), race (non-Hispanic White, Non-Hispanic Black, Hispanic, other race/ethnicity), education levels (some college or less, college graduate, postgraduate)

^e^Model 2 was additionally controlled with a family history of colorectal cancer (no, yes or possibly), history of colon comorbidity (no, yes), history of diverticulitis or diverticulosis (no, yes), history of colorectal polyp (no, yes), history of diabetes (no, yes), history of aspirin use (no, yes), total energy intake (continuous), body mass index at baseline (continuous), smoking status (never, current, former), pack-years of cigarettes (continuous), alcohol consumption (continuous), and physical activity level (continuous)

^f^Model 3 was additionally controlled with a carbohydrate quality index (continuous)

Subgroup analyses were consistent with the overall null findings between LCDs and CRC incidence (Additional file 1: Table S7). Additionally, sensitivity analyses showed that the lack of significant associations between CQI and CRC incidence or mortality remained unchanged (data not shown).

## Discussion

In this prospective cohort study of US adults, we found that higher carbohydrate quality as assessed by CQI was significantly associated with reduced CRC incidence and mortality. These inverse associations remained robust in the subgroup and sensitivity analyses. In subsite-specific analyses, higher CQI was associated with a 35% lower incidence of distal colon cancer and a 42% lower incidence of rectal cancer. Higher CQI was also associated with a 73% lower risk of dying from rectum cancer. In contrast, lower carbohydrate quantity as measured by LCDs showed no significant correlations with CRC outcomes, suggesting reduced carbohydrate quantity alone may not lower CRC burden in the American population. Overall, our findings indicate carbohydrate quality instead of quantity may be an important protective factor against CRC, particularly for distal colon and rectal cancers.

The CQI emphasizes diets high in dietary fiber; low in glycemic index; with a higher ratio of solid to total carbohydrates, indicating restricted alcohol and sugar-sweetened beverages; and a higher ratio of whole to total grains [10]. These interconnected diet quality factors may contribute to reduced CRC burden through several mechanisms. Specifically, the high intakes of dietary fiber increase stool bulk and accelerate colonic transit, reducing mucosal contact time with carcinogens and tumor promoters [24]. Colonic fermentation of fiber also produces short-chain fatty acids like butyrate that confer anti-inflammatory and anticarcinogenic effects [25]. Furthermore, the lower glycemic index and restricted sugar-sweetened beverages mitigate hyperinsulinemia and obesity, insulin resistance, and chronic inflammation, which can reduce CRC risk [26–28]. Restricting alcohol may suppress acetaldehyde production by colonic bacteria, thus lowering DNA damage, resisting epigenetic dysregulation, and inhibiting colorectal tumorigenesis [29]. Higher whole grain consumption provides abundant antioxidants, vitamins, minerals, and phytochemicals that counter oxidative stress and inflammation driving neoplastic changes [30]. In summary, the synergistic actions of high-quality carbohydrates on critical risk factors and pathways, from colonic milieu to systemic metabolism, may contribute to their observed strong inverse associations with CRC.

In this study, the protective associations between higher CQI and reduced CRC incidence and mortality were primarily observed for distal colon and rectum cancer rather than proximal colon cancer. This aligns with previous evidence suggesting stronger inverse diet-cancer relationships in the distal colon versus rectum than in the proximal colon regions [31, 32]. Compared to the proximal colon, the distal colon and particularly the rectum have greater carcinogen exposure due to prolonged transit times and fecal retention [31, 33]. The higher dietary fiber emphasized by CQI may confer particular benefits in the distal colorectum by accelerating transit, reducing genotoxic contact, enhancing butyrate production [25], and suppressing chronic inflammation [27]. The specific benefits of carbohydrate quality for distal colon and rectal cancer warrant further research on potential diet-microbiome-metabolite interactions along the colorectum. Elucidating such regional specificity of diet-cancer associations can inform targeted preventive strategies.

To the best of our knowledge, only one small case–control study from Iran (71 CRC cases and 142 controls) has reported associations between higher CQI and lower CRC risk (T3-OR = 0.15; 95% CI: 0.06–0.39), as well as an inverse link between LCDs and CRC incidence (T3-OR = 0.28; 95% CI: 0.10–0.82) [11]. Our results are partly consistent with this case–control study in terms of the inverse association between CRC incidence and CQI, but differ on the LCDs finding. However, the limited sample size and retrospective design limit the ability to draw definitive conclusions from this study. Notably, large cohort studies have found more nuanced relationships between LCDs and CRC outcomes. One cohort study from Singapore reported higher animal-based LCDs were associated with increased CRC risk [8], while another from the American cohort found plant-based LCDs linked to the decreased CRC-related mortality [9]. Importantly, LCDs based solely on reduced carbohydrate quantity did not show significant associations with CRC incidence and related mortality in both of the above cohort analyses [8, 9]. This aligns with our finding of LCDs, indicating overall carbohydrate restriction without consideration of food sources may not influence CRC risk.

Interestingly, our subgroup findings indicate that the protective association between higher CQI and reduced CRC incidence was only evident among individuals with lower LCDs (HR _Quartile 4 *vs.* Quartile 1_: 0.69; 95% CI: 0.54, 0.87; *P* = 0.001 for trend), but not those with higher LCDs (HR _Quartile 4 *vs.* Quartile 1_: 0.91; 95% CI: 0.70, 1.19; *P* = 0.484 for trend), although statistical tests for interaction did not meet significance thresholds (*P*_-interactions_ > 0.05). This novel result highlights that simply restricting overall carbohydrate amount may attenuate the protective effects of high-quality carbohydrate diets emphasized by CQI. In contrast, higher LCDs were not associated with decreased CRC risk across strata of higher or lower CQI. Overall, these data suggest that maintaining higher carbohydrate quality as reflected by CQI may be relevant for lowering CRC risk instead of restrictive carbohydrate intake, among individuals with more rather than less carbohydrate consumption. Taken together, our results provide novel preliminary evidence on the interplay between carbohydrate quality and quantity in shaping CRC susceptibility. Further research is warranted to clarify the optimal balance between carbohydrate amount versus quality for CRC prevention.

This study possesses several notable strengths, setting it apart from previous research. Firstly, it stands as the first large-scale, prospective investigation to concurrently explore the correlations between CQI and LCDs with both CRC incidence and mortality within a US cohort. This novel approach offers valuable insights into the role of carbohydrate quality and quantity in influencing CRC outcomes. Secondly, the extensive follow-up period and the inclusion of a large sample size significantly bolstered the statistical power of our study and increased the generalizability of the findings to similar populations. Thirdly, to minimize any potential biases, we conducted meticulous adjustments for an array of confounding factors in our analyses. Moreover, we performed a special subgroup analysis that yielded unique preliminary evidence regarding the interaction between CQI and LCDs in influencing CRC incidence. This exploration suggests that adhering to a higher CQI may not confer a significant benefit in reducing the risk of CRC in individuals with higher LCDs (i.e., those with lower carbohydrate intake). This observation raises intriguing questions about the potential complex interplay between carbohydrate quality and quantity in relation to CRC outcomes, warranting further investigation. Additionally, we performed sensitivity analyses to test the robustness of our results across various assumptions, reinforcing the reliability of our findings.

However, some limitations should be acknowledged. Firstly, our assessment of dietary intake was conducted only at baseline using DHQ, without capturing potential changes over time. While baseline diet assessments reasonably reflect habitual long-term intake patterns based on nutritional tenets [34]. Hence, the single DHQ measure provided valid representations of participants' customary diets before and during the study. Secondly, the possibility of residual confounding from unmeasured factors cannot be entirely excluded, as is the case with most observational studies. Thirdly, given the study's focus on older adults in the US, caution should be exercised when generalizing the results to other age groups or different countries, as dietary and lifestyle factors may differ. Lastly, as with any observational design, causal inferences concerning the identified diet-cancer associations must be interpreted with caution, warranting the need for future interventional studies to establish causality definitively.

## Conclusions

This uniquely comprehensive investigation in older Americans provides strong evidence that emphasizing carbohydrate quality over quantity may confer protection against CRC, particularly for distal colon and rectal tumors. These thought-provoking findings lay the groundwork for additional research to further elucidate relationships between carbohydrate characteristics and regional CRC susceptibility. Besides, future studies should be conducted to explore this association in other populations to verify the generalizability of these findings.

### Supplementary Information

**Additional file 1: Table S1.** Construction of Carbohydrate quality index and Low-carbohydrate diet score. **Table S2.** Distribution of covariates with missing data before and after imputation. **Table S3.** Subgroup analyses on the association between CQI and CRC incidence. **Table S4.** Sensitivity analyses on the between CQI and CRC incidence. **Table S5.** Sensitivity analyses on the between CQI and CRC mortality. **Table S6.** Association between LCDs and the CRC mortality according to main anatomic location. **Table S7.** Subgroup analyses on the association between LCDs and CRC incidence.

## Data Availability

The raw data used in this article is not available because of the National Cancer Institute's data policy. Access to the dataset should contact the National Cancer Institute by mail. For information on how to submit an application for gaining access to PLCO data, please follow the instructions at https://cdas.cancer.gov/plco/.

## Abbreviations

BMI: Body mass index
BQ: Baseline Questionnaire
CI: Confidence interval
CQI: Carbohydrate quality index
CRC: Colorectal cancer
DHQ: Dietary History Questionnaire
HR: Hazard ratio
NCI: National Cancer Institute
PLCO: Prostate lung colorectal and ovarian
SD: Standard deviation
SQX: Supplemental questionnaire
LCDs: Low-carbohydrate diet scores
