# Supplementary material for: Carbohydrate quality, not quantity, linked to reduced colorectal cancer incidence and mortality in US populations: evidence from a prospective study
Source: BMC Med. 2024 Mar 5;22:97. doi: 10.1186/s12916-024-03325-y (PMC10916156; doi:10.1186/s12916-024-03325-y)
Supplement: Supplementary file 1 — Additional file 1: Table S1. Construction of Carbohydrate quality index and Low-carbohydrate diet score. Table S2. Distribution of covariates with missing data before and after imputation. Table S3. Subgroup analyses on the association between CQI and CRC incidence. Table S4. Sensitivity analyses on the between CQI and CRC incidence. Table S5. Sensitivity analyses on the between CQI and CRC mortality. Table S6. Association between LCDs and the CRC mortality according to main anatomic location. Table S7. Subgroup analyses on the association between LCDs and CRC incidence. [file 12916_2024_3325_MOESM1_ESM.docx]

***Supplementary Material***

**Carbohydrate Quality, not Quantity, linked to reduced Colorectal Cancer incidence and mortality in US Populations: Evidence from a prospective study**

**Yi Xiao^1, #^, Ling Xiang^2, #, †^, Yahui Jiang^1^, Yunhao Tang^1^, Haitao Gu^1^, Yaxu Wang^1, †^, Linglong Peng^1,^ ^†^**

^1^ Department of Gastrointestinal Surgery, The Second Affiliated Hospital of Chongqing Medical University, Chongqing, China.

^2^ Department of Clinical Nutrition, The Second Affiliated Hospital of Chongqing Medical University, Chongqing, China.

**†** Correspondence authors: Ling Xiang (Department of Clinical Nutrition), Yaxu Wang and Linglong Peng (Department of Gastrointestinal Surgery), The Second Affiliated Hospital of Chongqing Medical University, No.288 Tianwen Avenue, Nan'an District, Chongqing, 400010, China. fax: +86 023 6288 7521. E-mail: 306359@hospital.cqmu.edu.cn (Ling Xiang); [300897@hospital.cqmu.edu.cn](mailto:300897@hospital.cqmu.edu.cn) (Yaxu Wang); penglinglong_cqmu@cqmu.edu.cn (Linglong Peng)

^#^ These authors contributed equally: Yi Xiao, Ling Xiang.

Table of contents

**Table S1**. Construction of Carbohydrate quality index and Low-carbohydrate diet score.

**Table S2**. Distribution of covariates with missing data before and after imputation.

**Table S3**. Subgroup analyses on the association between **CQI** and CRC incidence.

**Table S4**. Sensitivity analyses on the between CQI and CRC incidence.

**Table S5**. Sensitivity analyses on the between CQI and CRC mortality.

**Table S6**. Association between LCDs and the CRC mortality according to main anatomic location.

**Table S7**. Subgroup analyses on the association between LCDs and CRC incidence.

**Table S1.** Construction of **Carbohydrate quality index** and **Low-carbohydrate diet score**.

| **Components** | **Range (points)** | **Minimum index ^1^** | **Maximum index** |
| --- | --- | --- | --- |
| **Carbohydrate quality index** |  |  |  |
| 1. Dietary fiber intake (g/d) | 1–5 | Minimum intake (first quintile) | Maximum intake (fifth quintile) |
| 1. Glycemic index | 1–5 | Maximum index (fifth quintile) | Minimum index (first quintile) |
| 1. Ratio of whole-grain: total-grain carbohydrates | 1–5 | Minimum value of this ratio (first quintile) | Maximum value of this ratio (fifth quintile) |
| 1. Ratio solid carbohydrates:(solid carbohydrates + liquid carbohydrates) | 1–5 | Minimum value of this ratio (first quintile) | Maximum value of this ratio (fifth quintile) |
| Total index (range) | 4-20 |  |  |
| **Low-carbohydrate diet score** |  |  |  |
| 1. Carbohydrate intake (% E) | 0–10 | Maximum intake (first 1/11) | Minimum intake (last 1/11) |
| 1. Fat intake (% E) | 0–10 | Minimum intake (last 1/11) | Maximum intake (first 1/11) |
| 1. Protein intake (% E) | 0–10 | Minimum intake (last 1/11) | Maximum intake (first 1/11) |
| Total score (range) | 0–30 |  |  |

Abbreviations: %E, the percentage of total energy intake.

**1:** Proportional dietary indices were computed for intakes ranging between the maximum and minimum criteria.

**Table S2.** Distribution of covariates with missing data before and after imputation

| Variable | Before imputation | After imputation | Number (%) with missing data |
| --- | --- | --- | --- |
| Educational level: Some college or less | 64708 (63.8%) | 64912 (63.8%) | 204 (0.20%) |
| Body mass index at baseline (kg/m^2^) | 27.2 (4.8) | 27.2 (4.8) | 1348 (1.33%) |
| Smoking status: Never | 48538 (47.7%) | 48558 (47.7%) | 20 (0.02%) |
| Pack-years of cigarettes | 17.8 (26.7) | 17.6 (26.6) | 1163 (1.14%) |
| Aspirin user: No | 53476 (52.8%) | 53920 (53.0%) | 444 (0.44%) |
| Family history of colorectal cancer: No | 88114 (87.3%) | 88895 (87.4%) | 781 (0.77%) |
| History of diabetes: No | 94358 (93.3%) | 94896 (93.3%) | 538 (0.53%) |
| History of diverticulitis or diverticulosis: No | 94231 (93.3%) | 94874 (93.3%) | 643 (0.63%) |
| History of colon comorbidity: No | 99441 (98.7%) | 100338 (98.7%) | 897 (0.88%) |
| History of colorectal polyp: No | 94306 (93.3%) | 94932 (93.4%) | 626 (0.62%) |
| Physical activity level (min/week) | 125.0 (123.6) | 125.3 (108.3) | 26036 (25.60%) |

Values are mean (standard deviation) or counts (percentage) as indicated.

|  |  |  | **CQI**, HR (95% CI) | | | |  |  |
| --- | --- | --- | --- | --- | --- | --- | --- | --- |
| Subgroup variable | Cases, n | Person-years | Quartile 1 | Quartile 2 | Quartile 3 | Quartile 4 | ***P* _trend_** ^1^ | ***P* _interaction_** ^2^ |
| **Age (years)** |  |  |  |  |  |  |  | 0.908 |
| ≤65 | 396 | 468,405 | 1.00 (reference) | 0.86 (0.66, 1.12) | 0.82 (0.62, 1.09) | 0.77 (0.57, 1.04) | 0.057 |  |
| >65 | 689 | 427,635 | 1.00 (reference) | 0.93 (0.75, 1.14) | 0.94 (0.76, 1.16) | 0.86 (0.69, 1.07) | 0.190 |  |
| **Sex** |  |  |  |  |  |  |  | 0.971 |
| Male | 601 | 430,685 | 1.00 (reference) | 0.90 (0.72, 1.12) | 0.85 (0.68, 1.07) | 0.80 (0.63, 1.01) | 0.056 |  |
| Female | 484 | 465,357 | 1.00 (reference) | 0.88 (0.69, 1.12) | 0.91 (0.71, 1.17) | 0.80 (0.61, 1.05) | 0.109 |  |
| **Smoking status** |  |  |  |  |  |  |  | 0.244 |
| Never | 481 | 434,396 | 1.00 (reference) | 0.74 (0.58, 0.95) | 0.82 (0.64, 1.05) | 0.72 (0.56, 0.94) | 0.016 |  |
| Current/Former | 604 | 461,645 | 1.00 (reference) | 1.02 (0.83, 1.27) | 0.93 (0.74, 1.17) | 0.87 (0.68, 1.10) | 0.240 |  |
| **BMI at baseline (kg/m^2^)** |  |  |  |  |  |  |  | 0.134 |
| ＜30 | 823 | 696,334 | 1.00 (reference) | 0.92 (0.76, 1.11) | 0.85 (0.70, 1.03) | 0.73 (0.59, 0.89) | 0.002 |  |
| ≥30 | 262 | 199,707 | 1.00 (reference) | 0.78 (0.56, 1.10) | 0.93 (0.66, 1.31) | 1.05 (0.74, 1.48) | 0.899 |  |
| **Diabetes history** |  |  |  |  |  |  |  | 0.058 |
| No | 983 | 840,332 | 1.00 (reference) | 0.92 (0.78, 1.09) | 0.89 (0.74, 1.06) | 0.86 (0.72, 1.04) | 0.101 |  |
| Yes | 102 | 55,710 | 1.00 (reference) | 0.66 (0.39, 1.10) | 0.79 (0.47, 1.33) | 0.34 (0.17, 0.68) | 0.003 |  |
| **Aspirin use regularly** |  |  |  |  |  |  |  | 0.634 |
| No | 598 | 478,883 | 1.00 (reference) | 0.89 (0.72, 1.11) | 0.87 (0.70, 1.10) | 0.77 (0.60, 0.98) | 0.032 |  |
| Yes | 487 | 417,158 | 1.00 (reference) | 0.89 (0.70, 1.14) | 0.88 (0.68, 1.13) | 0.84 (0.65, 1.09) | 0.175 |  |
| **Energy intake from diet (kcal/day)** |  |  |  |  |  |  |  | 0.393 |
| ≤median ^3^ | 542 | 448,372 | 1.00 (reference) | 0.98 (0.79, 1.22) | 0.84 (0.66, 1.07) | 0.87 (0.67, 1.13) | 0.199 |  |
| >median | 543 | 447,669 | 1.00 (reference) | 0.79 (0.62, 1.02) | 0.89 (0.71, 1.13) | 0.76 (0.60, 0.96) | 0.047 |  |
| **LCDs** |  |  |  |  |  |  |  | 0.301 |
| ≤median ^4^ | 597 | 476,782 | 1.00 (reference) | 0.88 (0.71, 1.10) | 0.79 (0.63, 0.99) | 0.69 (0.54, 0.87) | 0.001 |  |
| >median | 488 | 419,260 | 1.00 (reference) | 0.88 (0.69, 1.12) | 0.95 (0.74, 1.22) | 0.91 (0.70, 1.19) | 0.484 |  |

**Table S3**. Subgroup analyses on the association between **CQI** and CRC incidence.

Abbreviations: CQI, carbohydrate quality index; CRC, colorectal cancer; HR, hazard ratio; CI, confidence interval; LCDs, Low-carbohydrate diet score.

**1:** Trend test was performed using median value of each diet score quintile as a continuous variable.

**2**: P value for interaction was estimated using the likelihood ratio test comparing the model with and without the interaction term of the paleolithic diet score and the respective stratification variable.

**3**: The median of dietary energy intake in this study is 1607 kcal/day.

**4**: The median of LCDs in this study is 15.

Hazard ratios were adjusted for age (continuous), sex (male, female), race (Non-Hispanic White, Non-Hispanic Black, Hispanic, other race/ethnicity), education levels (some college or less, college graduate, postgraduate), family history of colorectal cancer (no, yes or possibly), history of colon comorbidity (no, yes), history of diverticulitis or diverticulosis (no, yes), history of colorectal polyp (no, yes), history of diabetes (no, yes), history of aspirin use (no, yes), total energy intake (continuous), body mass index at baseline (continuous), smoking status (never, current, former), pack-years of cigarettes (continuous), alcohol consumption (continuous), physical activity level (continuous), and LCDs (continuous).

**Table S4**. Sensitivity analyses on the between **CQI** and CRC incidence.

| Categories | **participants** | **cases** | HR Quartile 4 vs. Quartile 1 (95% CI) ^1^ | P_-trend_ |
| --- | --- | --- | --- | --- |
| Primary analysis | 101694 | 1085 | 0.80 (0.67, 0.96) | 0.012 |
| Excluded participants with extreme energy intake ^2^ | 100220 | 1063 | 0.81 (0.68, 0.97) | 0.020 |
| Excluded participants with extreme BMI ^3^ | 99703 | 1065 | 0.79 (0.66, 0.94) | 0.007 |
| Excluded cases observed within the first 1 years of follow-up | 101579 | 970 | 0.77 (0.64, 0.93) | 0.006 |
| Excluded cases observed within the first 2 years of follow-up | 101456 | 847 | 0.73 (0.59, 0.89) | 0.001 |
| Excluded participants with colon comorbidity ^4^ | 100338 | 1069 | 0.81 (0.68, 0.97) | 0.017 |
| Excluded participants with colorectal polyp | 94932 | 994 | 0.80 (0.67, 0.96) | 0.015 |
| Excluded participants with family history of colorectal cancer | 88895 | 922 | 0.83 (0.68, 1.00) | 0.036 |
| Further adjusted for carbohydrate intake (% E) ^5^ | 101694 | 1085 | 0.79 (0.66, 0.94) | 0.007 |
| Further adjusted for several dietary factors ^6^ | 101694 | 1085 | 0.82 (0.68, 0.98) | 0.026 |

Abbreviations: HR, hazard ratio; CI, confidence interval;

**1**: Hazard ratios were adjusted for age (continuous), sex (male, female), race (Non-Hispanic White, Non-Hispanic Black, Hispanic, other race/ethnicity), education levels (some college or less, college graduate, postgraduate), family history of colorectal cancer (no, yes or possibly), history of colon comorbidity (no, yes), history of diverticulitis or diverticulosis (no, yes), history of colorectal polyp (no, yes), history of diabetes (no, yes), history of aspirin use (no, yes), total energy intake (continuous), body mass index at baseline (continuous), smoking status (never, current, former), pack-years of cigarettes (continuous), alcohol consumption (continuous), physical activity level (continuous), and LCDs (continuous).

**2**: The extreme energy intake was defined as >4000 kcal/day or <500 kcal/day;

**3**: The extreme BMI (baseline) was defined as top 1% or bottom 1% in the included population.

**4**: The colon comorbidities including ulcerative colitis, Crohn's disease, Gardner's syndrome, and familial polyposis.

**5**: The carbohydrate intake (% E) was used in the fully adjusted model instead of Low-carbohydrate diet score.

**6**: The dietary factors including the energy-adjusted consumption of dietary calcium, calcium from supplements, energy-adjusted average daily red meat, and total folate (combining dietary folate and folate from supplements).

**Table S5**. Sensitivity analyses on the between **CQI** and CRC mortality.

| Categories | **participants** | **cases** | HR Quartile 4 vs. Quartile 1 (95% CI) ^1^ | P_-trend_ |
| --- | --- | --- | --- | --- |
| Primary analysis | 101694 | 311 | 0.61 (0.44, 0.86) | 0.004 |
| Excluded participants with extreme energy intake ^2^ | 100220 | 300 | 0.64 (0.45, 0.91) | 0.013 |
| Excluded participants with extreme BMI ^3^ | 99703 | 310 | 0.60 (0.43, 0.85) | 0.003 |
| Excluded cases observed within the first 1 years of follow-up | 101690 | 307 | 0.63 (0.44, 0.88) | 0.006 |
| Excluded cases observed within the first 2 years of follow-up | 101680 | 297 | 0.59 (0.42, 0.84) | 0.003 |
| Excluded participants with colon comorbidity ^4^ | 100338 | 307 | 0.61 (0.44, 0.86) | 0.004 |
| Excluded participants with colorectal polyp | 94932 | 283 | 0.62 (0.43, 0.88) | 0.005 |
| Excluded participants with family history of colorectal cancer | 88895 | 262 | 0.69 (0.48, 1.00) | 0.041 |
| Further adjusted for carbohydrate intake (% E) ^5^ | 101694 | 311 | 0.60 (0.43, 0.85) | 0.003 |
| Further adjusted for several dietary factors ^6^ | 101694 | 311 | 0.62 (0.44, 0.88) | 0.006 |

Abbreviations: HR, hazard ratio; CI, confidence interval;

**1**: Hazard ratios were adjusted for age (continuous), sex (male, female), race (Non-Hispanic White, Non-Hispanic Black, Hispanic, other race/ethnicity), education levels (some college or less, college graduate, postgraduate), family history of colorectal cancer (no, yes or possibly), history of colon comorbidity (no, yes), history of diverticulitis or diverticulosis (no, yes), history of colorectal polyp (no, yes), history of diabetes (no, yes), history of aspirin use (no, yes), total energy intake (continuous), body mass index at baseline (continuous), smoking status (never, current, former), pack-years of cigarettes (continuous), alcohol consumption (continuous), physical activity level (continuous), and LCDs (continuous).

**2**: The extreme energy intake was defined as >4000 kcal/day or <500 kcal/day;

**3**: The extreme BMI (baseline) was defined as top 1% or bottom 1% in the included population.

**4**: The colon comorbidities including ulcerative colitis, Crohn's disease, Gardner's syndrome, and familial polyposis.

**5**: The carbohydrate intake (% E) was used in the fully adjusted model instead of Low-carbohydrate diet score.

**6**: The dietary factors including the energy-adjusted consumption of dietary calcium, calcium from supplements, energy-adjusted average daily red meat, and total folate (combining dietary folate and folate from supplements).

| **Outcome** | **LCD**s, HR (95% CI) | | | |  | Continuous (per SD increment) |
| --- | --- | --- | --- | --- | --- | --- |
|  | Quartile 1 (lowest) | Quartile 2 | Quartile 3 | Quartile 4 (highest) | P for trend ^1^ |  |
| Mean (SD) value of **LCDs** | 6.4 (3.0) | 13.0 (1.4) | 18.0 (1.4) | 24.3 (2.6) |  |  |
| Person-years | 436,228 | 373,751 | 357,949 | 364,568 |  |  |
| **Overall** ^2^ |  |  |  |  |  |  |
| Cases, n | 91 | 74 | 68 | 78 |  |  |
| Incidence rate (95% CI) ^3^ | 0.21 (0.17, 0.26) | 0.20 (0.16, 0.25) | 0.19 (0.15, 0.24) | 0.21 (0.17, 0.27) |  |  |
| Model 1 ^4^ | 1.00 (reference) | 0.98 (0.72, 1.34) | 0.96 (0.70, 1.32) | 1.15 (0.85, 1.57) | 0.439 | 1.03 (0.92, 1.15) |
| Model 2 ^5^ | 1.00 (reference) | 0.96 (0.70, 1.31) | 0.93 (0.67, 1.28) | 1.06 (0.77, 1.47) | 0.788 | 0.99 (0.88, 1.11) |
| Model 3 ^6^ | 1.00 (reference) | 0.96 (0.70, 1.31) | 0.91 (0.66, 1.25) | 1.02 (0.74, 1.42) | 0.982 | 0.98 (0.87, 1.10) |
| **Proximal colon** |  |  |  |  |  |  |
| Cases, n | 61 | 41 | 37 | 42 |  |  |
| Incidence rate (95% CI) ^3^ | 0.14 (0.11, 0.18) | 0.11 (0.08, 0.15) | 0.10 (0.07, 0.14) | 0.12 (0.09, 0.16) |  |  |
| Model 1 ^4^ | 1.00 (reference) | 0.81 (0.54, 1.21) | 0.79 (0.52, 1.19) | 0.94 (0.63, 1.41) | 0.656 | 0.96 (0.83, 1.12) |
| Model 2 ^5^ | 1.00 (reference) | 0.79 (0.53, 1.19) | 0.77 (0.51, 1.17) | 0.89 (0.59, 1.35) | 0.497 | 0.93 (0.80, 1.09) |
| Model 3 ^6^ | 1.00 (reference) | 0.79 (0.53, 1.18) | 0.76 (0.50, 1.15) | 0.87 (0.57, 1.33) | 0.430 | 0.92 (0.79, 1.08) |
| **Distal colon** |  |  |  |  |  |  |
| Cases, n | 14 | 18 | 20 | 19 |  |  |
| Incidence rate (95% CI) ^3^ | 0.03 (0.02, 0.05) | 0.05 (0.03, 0.08) | 0.06 (0.04, 0.09) | 0.05 (0.03, 0.08) |  |  |
| Model 1 ^4^ | 1.00 (reference) | 1.53 (0.76, 3.08) | 1.78 (0.89, 3.54) | 1.73 (0.86, 3.50) | 0.109 | 1.15 (0.90, 1.46) |
| Model 2 ^5^ | 1.00 (reference) | 1.47 (0.72, 2.97) | 1.64 (0.81, 3.31) | 1.49 (0.71, 3.10) | 0.282 | 1.07 (0.83, 1.37) |
| Model 3 ^6^ | 1.00 (reference) | 1.46 (0.72, 2.96) | 1.60 (0.79, 3.23) | 1.43 (0.68, 2.98) | 0.342 | 1.05 (0.82, 1.35) |
| **Rectum** |  |  |  |  |  |  |
| Cases, n | 16 | 13 | 10 | 15 |  |  |
| Incidence rate (95% CI) ^3^ | 0.04 (0.02, 0.06) | 0.03 (0.02, 0.06) | 0.03 (0.02, 0.05) | 0.04 (0.02, 0.07) |  |  |
| Model 1 ^4^ | 1.00 (reference) | 1.01 (0.48, 2.11) | 0.81 (0.37, 1.80) | 1.22 (0.59, 2.51) | 0.721 | 1.06 (0.81, 1.39) |
| Model 2 ^5^ | 1.00 (reference) | 1.01 (0.48, 2.12) | 0.81 (0.36, 1.82) | 1.19 (0.56, 2.55) | 0.773 | 1.04 (0.78, 1.39) |
| Model 3 ^6^ | 1.00 (reference) | 1.01 (0.48, 2.13) | 0.78 (0.35, 1.76) | 1.13 (0.53, 2.41) | 0.896 | 1.02 (0.77, 1.35) |

**Table S6.** Association between **LCD**s and the CRC mortality according to main anatomic location.

Abbreviations: CQI, carbohydrate quality index; CRC, colorectal cancer; HR, hazard ratio; CI, confidence interval, SD, standard deviation.

**1**: Trend test was performed using median value of each diet score quintile as a continuous variable.

**2**: Including 5 death cases related to CRC with an unknown anatomic location.

**3**: Incidence rate was calculated per 1000 person-years.

**4**: Model 1 was controlled with age (continuous), sex (male, female), race (Non-Hispanic White, Non-Hispanic Black, Hispanic, other race/ethnicity), education levels (some college or less, college graduate, postgraduate).

**5**: Model 2 was additionally controlled with family history of colorectal cancer (no, yes or possibly), history of colon comorbidity (no, yes), history of diverticulitis or diverticulosis (no, yes), history of colorectal polyp (no, yes), history of diabetes (no, yes), history of aspirin use (no, yes), total energy intake (continuous), body mass index at baseline (continuous), smoking status (never, current, former), pack-years of cigarettes (continuous), alcohol consumption (continuous), and physical activity level (continuous).

**6**: Model 3 was additionally controlled with carbohydrate quality index (continuous).

**Table S7**. Subgroup analyses on the association between **LCD**s and CRC incidence.

|  |  |  | **LCDs**, HR (95% CI) | | | |  |  |
| --- | --- | --- | --- | --- | --- | --- | --- | --- |
| Subgroup variable | Cases, n | Person-years | Quartile 1 | Quartile 2 | Quartile 3 | Quartile 4 | ***P* _trend_** ^1^ | ***P* _interaction_** ^2^ |
| **Age (years)** |  |  |  |  |  |  |  | 0.976 |
| ≤65 | 396 | 468,405 | 1.00 (reference) | 1.01 (0.76, 1.34) | 0.95 (0.71, 1.27) | 0.96 (0.72, 1.27) | 0.697 |  |
| >65 | 689 | 427,635 | 1.00 (reference) | 0.99 (0.81, 1.20) | 0.89 (0.72, 1.10) | 0.88 (0.70, 1.10) | 0.184 |  |
| **Sex** |  |  |  |  |  |  |  | 0.971 |
| Male | 601 | 430,685 | 1.00 (reference) | 1.05 (0.84, 1.32) | 0.98 (0.78, 1.24) | 0.97 (0.76, 1.23) | 0.714 |  |
| Female | 484 | 465,357 | 1.00 (reference) | 0.96 (0.76, 1.21) | 0.85 (0.66, 1.10) | 0.90 (0.69, 1.17) | 0.284 |  |
| **Smoking status** |  |  |  |  |  |  |  | 0.910 |
| Never | 481 | 434,396 | 1.00 (reference) | 0.74 (0.58, 0.95) | 0.82 (0.64, 1.05) | 0.72 (0.56, 0.94) | 0.378 |  |
| Current/Former | 604 | 461,645 | 1.00 (reference) | 1.02 (0.83, 1.27) | 0.93 (0.74, 1.17) | 0.87 (0.68, 1.10) | 0.456 |  |
| **BMI at baseline (kg/m^2^)** |  |  |  |  |  |  |  | 0.167 |
| ＜30 | 823 | 696,334 | 1.00 (reference) | 1.06 (0.89, 1.28) | 1.00 (0.82, 1.21) | 0.93 (0.75, 1.14) | 0.467 |  |
| ≥30 | 262 | 199,707 | 1.00 (reference) | 0.79 (0.55, 1.12) | 0.68 (0.47, 0.98) | 0.89 (0.64, 1.25) | 0.500 |  |
| **Diabetes history** |  |  |  |  |  |  |  | 0.732 |
| No | 983 | 840,332 | 1.00 (reference) | 1.02 (0.86, 1.21) | 0.92 (0.77, 1.10) | 0.96 (0.79, 1.15) | 0.450 |  |
| Yes | 102 | 55,710 | 1.00 (reference) | 0.75 (0.41, 1.38) | 0.83 (0.46, 1.48) | 0.65 (0.36, 1.17) | 0.191 |  |
| **Aspirin use regularly** |  |  |  |  |  |  |  | 0.652 |
| No | 598 | 478,883 | 1.00 (reference) | 1.04 (0.83, 1.28) | 0.87 (0.69, 1.10) | 0.92 (0.72, 1.17) | 0.302 |  |
| Yes | 487 | 417,158 | 1.00 (reference) | 0.95 (0.74, 1.22) | 0.96 (0.75, 1.24) | 0.93 (0.71, 1.21) | 0.616 |  |
| **Energy intake from diet (kcal/day)** |  |  |  |  |  |  |  | 0.384 |
| ≤median ^3^ | 542 | 448,372 | 1.00 (reference) | 0.91 (0.73, 1.13) | 0.86 (0.67, 1.09) | 0.98 (0.76, 1.25) | 0.587 |  |
| >median | 543 | 447,669 | 1.00 (reference) | 1.13 (0.88, 1.44) | 1.00 (0.78, 1.29) | 0.93 (0.72, 1.20) | 0.386 |  |
| **CQI** |  |  |  |  |  |  |  | 0.217 |
| ≤median ^4^ | 622 | 492,679 | 1.00 (reference) | 1.07 (0.86, 1.32) | 0.84 (0.67, 1.06) | 0.92 (0.73, 1.16) | 0.229 |  |
| >median | 463 | 403,363 | 1.00 (reference) | 0.92 (0.72, 1.18) | 1.03 (0.79, 1.33) | 0.96 (0.73, 1.27) | 0.932 |  |

Abbreviations: LCDs, Low-carbohydrate diet score; CRC, colorectal cancer; HR, hazard ratio; CI, confidence interval; CQI, carbohydrate quality index.

**1:** Trend test was performed using median value of each diet score quintile as a continuous variable.

**2**: P value for interaction was estimated using the likelihood ratio test comparing the model with and without the interaction term of the paleolithic diet score and the respective stratification variable.

**3**: The median of dietary energy intake in this study is 1607 kcal/day.

**4**: The median of CQI score in this study is 12.

Hazard ratios were adjusted for age (continuous), sex (male, female), race (Non-Hispanic White, Non-Hispanic Black, Hispanic, other race/ethnicity), education levels (some college or less, college graduate, postgraduate), family history of colorectal cancer (no, yes or possibly), history of colon comorbidity (no, yes), history of diverticulitis or diverticulosis (no, yes), history of colorectal polyp (no, yes), history of diabetes (no, yes), history of aspirin use (no, yes), total energy intake (continuous), body mass index at baseline (continuous), smoking status (never, current, former), pack-years of cigarettes (continuous), alcohol consumption (continuous), physical activity level (continuous), and CQI score (continuous).
